# Diversity and Composition of Bacterial Community in Soils and Lake Sediments from an Arctic Lake Area

**DOI:** 10.3389/fmicb.2016.01170

**Published:** 2016-07-28

**Authors:** Neng Fei Wang, Tao Zhang, Xiao Yang, Shuang Wang, Yong Yu, Long Long Dong, Yu Dong Guo, Yong Xing Ma, Jia Ye Zang

**Affiliations:** ^1^Key Lab of Marine Bioactive Substances, First Institute of Oceanography, State Oceanic AdministrationQingdao, China; ^2^Institute of Medicinal Biotechnology, Chinese Academy of Medical SciencesBeijing, China; ^3^Chemical Engineering Institute, Qingdao UniversityQingdao, China; ^4^Polar Research Institute of ChinaShanghai, China; ^5^Department of Bioengineering and Biotechnology, Qingdao University of Science and TechnologyQingdao, China

**Keywords:** bacterial diversity and community composition, Arctic lake area, soils and lake sediments, high-throughput sequencing, geochemical factor

## Abstract

This study assessed the diversity and composition of bacterial communities within soils and lake sediments from an Arctic lake area (London Island, Svalbard). A total of 2,987 operational taxonomic units were identified by high-throughput sequencing, targeting bacterial 16S rRNA gene. The samples from four sites (three samples in each site) were significantly different in geochemical properties and bacterial community composition. Proteobacteria and Acidobacteria were abundant phyla in the nine soil samples, whereas Proteobacteria and Bacteroidetes were abundant phyla in the three sediment samples. Furthermore, Actinobacteria, Chlorobi, Chloroflexi, Elusimicrobia, Firmicutes, Gemmatimonadetes, Nitrospirae, Planctomycetes, Proteobacteria significantly varied in their abundance among the four sampling sites. Additionally, members of the dominant genera, such as *Clostridium*, *Luteolibacter*, *Methylibium*, *Rhodococcus*, and *Rhodoplanes*, were significantly different in their abundance among the four sampling sites. Besides, distance-based redundancy analysis revealed that pH (*p* < 0.001), water content (*p* < 0.01), ammonium nitrogen (${\mathrm{NH}}_{4}^{+}$-N, *p* < 0.01), silicate silicon (${\mathrm{SiO}}_{4}^{2-}$-Si, *p* < 0.01), nitrite nitrogen (${\mathrm{NO}}_{2}^{-}$-N, *p* < 0.05), organic carbon (*p* < 0.05), and organic nitrogen (*p* < 0.05) were the most significant factors that correlated with the bacterial community composition. The results suggest soils and sediments from a lake area in the Arctic harbor a high diversity of bacterial communities, which are influenced by many geochemical factors of Arctic environments.

## Introduction

Thaw lakes and ponds are major features of the Arctic landscape and can account for up to 90% of the total land surface area in the parts of the Arctic (Yoshikawa and Hinzman, 2003; Smol and Douglas, 2007; Pienitz et al., 2008). Over the past 100 years, the Arctic average temperatures have increased at about twice the global average rate (Solomon, 2007) and one consequence of the rising temperatures in the Arctic is the changes in the distribution and abundance of thaw lakes and ponds (Vincent et al., 2013). In some regions of the High Arctic, a few lakes and ponds are beginning to shrink, and some have even disappeared (Smith et al., 2005). In contrasts, in some regions of Low Arctic or Subarctic, some thaw lakes and ponds are growing in size because of permafrost thaw (Payette et al., 2004). Therefore, lakes and ponds are good sentinels of global climate change (Adrian et al., 2009).

Arctic microbes are sensitive to environmental changes in the Arctic ecosystem (Vincent, 2010) and thereby understanding the Arctic microbial community is of great importance to predict the response of the Arctic ecosystems to climate warming (Blaud et al., 2015a). As the key components of microbes, Bacteria and Archaea within soils or lake sediments may play important roles in driving biogeochemical cycles (e.g., carbon, nitrogen, and phosphorus) in the Arctic lake area. Hitherto, a few studies were carried out to provide a great deal of information on the bacterial diversity in the soils from various area types, such as diesel-contaminated soils (Yergeau et al., 2012), peat soils (Lipson et al., 2013), permafrost-affected soils (Gittel et al., 2014b), buried soils (Gittel et al., 2014a), vegetation-impacted soils (Shi et al., 2015), tundra tussock and shrub soils (Wallenstein et al., 2007). To the best of our knowledge, only two studies have yet been carried out to provide insight to the bacterial communities that inhabit soils or lake sediments from an Arctic lake area in the Canadian High Arctic (Steven et al., 2013; Stoeva et al., 2014). Moreover, the environmental factors which influence the bacterial community composition and function in the Arctic lake area are poorly understood.

Previous studies mainly focused on the diversity of cultured soil bacteria in the Arctic using traditional isolation methods (Master and Mohn, 1998) and conventional DNA-based molecular methods (e.g., DGGE, T-RFLP, Q-PCR, clone libraries) (Neufeld and Mohn, 2005; Wallenstein et al., 2007; Stoeva et al., 2014; Blaud et al., 2015b). Recently, bacterial diversity and community composition in the Arctic soils or lake sediments have been revealed by next-generation sequencing (Campbell et al., 2010; Yergeau et al., 2010, 2012, 2014; Mackelprang et al., 2011; Lipson et al., 2013; Gittel et al., 2014a,b; Kim et al., 2014; Koyama et al., 2014; Schostag et al., 2015; Shi et al., 2015). In the present study, we use high-throughput sequencing to investigate the bacterial communities in the soils and sediments from an Arctic lake area (Svalbard, High Arctic) and address the following questions: (1) what are bacterial diversity and community composition in the Arctic lake area; (2) what are differences in the bacterial taxonomic groups among different sampling sites in the Arctic lake area; (3) what are the key geochemical factors that determine the bacteria community composition in the Arctic lake area?

## Materials and Methods

### Study Sites and Sample Collection

**FIGURE 1:**
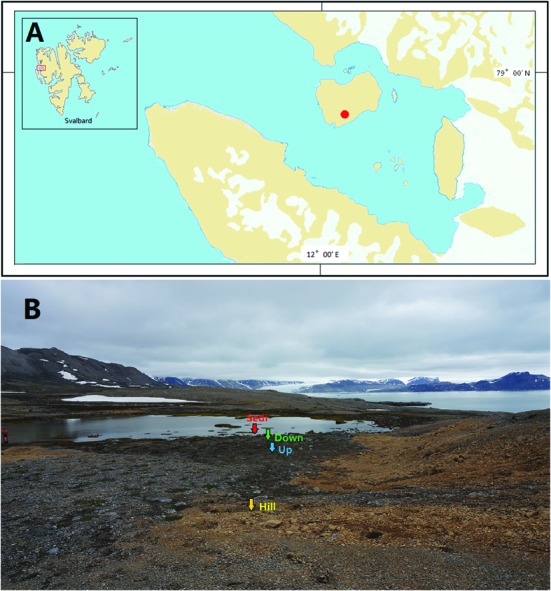
**Location (red dot; A) and image (B) of the sampling sites from an Arctic lake area in the present study**.

The studied area was located in the London Island of Konsfjorden, which is on the west coast of Spitsbergen, Svalbard archipelago (**Figure 1A**). During winter, this shallow lake (~2 m deep) can be frozen to the bottom, whereas during summer, it is supplied by meltwater from the lake ice and accumulated snow on the hillslope. Soils and sediments (about 50 g) were sampled from surface (5 cm) near each other (about 1 m apart) in triplicate at four sites (Hill, Up, Down, and Sedi; **Figure 1B**). Samples were collected using a sterile shovel and directly put into TWIRL’EM sterile sampling bags (Labplas Inc., Sainte-Julie, QC, Canada). Sampling occurred during China’s Arctic expedition in July 2014. A total of nine soil samples and three lake sediment samples were collected and directly put into sterile plastic sampling bags. Samples were placed in centrifuge tubes at -20°C in the Yellow River Station (China) for about 20 days and then taken to the home laboratory in China by plane. During the flights, samples were conserved in an incubator with frozen ice bags. In the home laboratory, soil samples were frozen at -80°C until nucleic acid extraction.

### Geochemical Properties of Soils and Lake Sediments

A total of nine geochemical properties were assessed, including pH, water content, organic carbon, organic nitrogen, ammonium nitrogen (${\mathrm{NH}}_{4}^{+}$-N), silicate silicon (${\mathrm{SiO}}_{4}^{2-}$-Si), nitrite nitrogen (${\mathrm{NO}}_{2}^{-}$-N), phosphate phosphorus (${\mathrm{PO}}_{4}^{3-}$-P), and nitrate nitrogen (${\mathrm{NO}}_{3}^{-}$-N) (**Table 1**). Adding 10 ml of distilled water to 4 g of soil/sediment, pH was measured by a pH electrode (PHS-3C, Shanghai REX Instrument Factory, Shanghai, China). Water content (10 g of each sample) was determined as gravimetric weight loss after drying the soil/sediment at 105°C until constant weight. The organic C and organic N were analyzed according to the following procedure (Hu et al., 2012). Samples (10 g of each sample) were dried at 50°C, homogenized and pulverized with an agate mortar. Approximately 0.5 g samples were thoroughly decalcified using 1 M hydrochloric acid. They were then subsequently rinsed with Milli-Q water for complete acid removal and dried overnight at 50°C. The carbonate-free total organic carbon and total organic nitrogen samples were analyzed using an elemental analyzer (EA3000, Euro Vector SpA, Milan, Italy). The amounts of organic C and organic N were indicated by percentage. The other five chemical properties (${\mathrm{SiO}}_{4}^{2-}$-Si, ${\mathrm{PO}}_{4}^{3-}$-P, ${\mathrm{NO}}_{3}^{-}$-N, ${\mathrm{NO}}_{2}^{-}$-N, and ${\mathrm{NH}}_{4}^{+}$-N; 5 g of each sample) were determined colorimetrically on a nutrient auto-analyzer (QuAAtro, SEAL, Germany; relative standard deviation < 5%; Liu et al., 2016), and the detection limits for ${\mathrm{SiO}}_{4}^{2-}$-Si, ${\mathrm{PO}}_{4}^{3-}$-P, ${\mathrm{NO}}_{3}^{-}$-N, ${\mathrm{NO}}_{2}^{-}$-N, and ${\mathrm{NH}}_{4}^{+}$-N were 0.030, 0.024, 0.015, 0.003, and 0.040 μmol L^-1^, respectively.

**Table 1:** Geochemical properties of 12 samples investigated in the present study.

Sample code	Type	Water content (%)	pH	Organic N (%)	Organic C (%)	${\mathrm{NH}}_{4}^{+}$-N (μg/g)	${\mathrm{SiO}}_{4}^{2-}$- Si (μg/g)	${\mathrm{NO}}_{2}^{-}$-N (μg/g)	${\mathrm{PO}}_{4}^{3-}$- P (μg/g)	${\mathrm{NO}}_{3}^{-}$-N (μg/g)
Hill.1	Soil	14.30	8.15	0.14	1.38	3.50	5.00	0.66	0.09	0.75
Hill.2	Soil	13.52	8.14	0.10	0.70	2.51	4.00	0.76	0.03	1.19
Hill.3	Soil	14.91	8.05	0.08	0.79	3.08	3.73	0.44	0.02	0.75
**Average**		**14.24^A^**	**8.11^B^**	**0.10**	**0.96**	**3.03^A^**	**4.25^A^**	**0.62^B^**	**0.05**	**0.89^AB^**
Up.1	Soil	10.14	8.01	0.10	0.76	1.48	4.59	0.76	0.04	1.40
Up.2	Soil	31.22	7.75	0.39	4.33	5.26	4.60	1.06	0.06	4.20
Up.3	Soil	10.00	8.22	0.07	0.54	2.50	4.31	1.01	0.03	2.21
**Average**		**17.12^A^**	**7.99^B^**	**0.18**	**1.88**	**3.08^A^**	**4.50^A^**	**0.94^B^**	**0.04**	**2.60^B^**
Down.1	Soil	10.45	8.51	0.09	0.86	1.67	8.90	0.57	0.01	1.01
Down.2	Soil	9.44	8.46	0.06	0.58	1.54	10.12	0.75	0.01	1.50
Down.3	Soil	8.68	8.44	0.10	0.44	1.96	9.84	0.69	ND	1.04
**Average**		**9.52^A^**	**8.47^C^**	**0.08**	**0.63**	**1.72^A^**	**9.62^A^**	**0.67^B^**	**0.01**	**1.18^AB^**
Sedi.1	Sediment	56.33	7.11	0.94	11.81	102.28	55.45	0.06	0.02	0.11
Sedi.2	Sediment	48.38	7.18	0.29	3.49	60.00	32.79	0.04	ND	0.07
Sedi.3	Sediment	52.06	7.12	0.24	2.79	110.22	68.88	0.10	1.71	0.19
**Average**		**52.26^B^**	**7.14^A^**	**0.49**	**6.03**	**90.84^B^**	**52.37^B^**	**0.06^A^**	**0.58**	**0.12^A^**

Statistical significance was assessed by one-way ANOVA followed by Tukey’s HSD test, and significant differences were accepted when *p* < 0.05 between the two groups. The letters A, B, and C were used to show statistically significant differences.

### DNA Extraction and PCR Amplification

DNA was extracted from an aliquot of 0.25 g soil/sediment from each sample, using a PowerSoil DNA Isolation Kit (MO BIO Laboratories, San Diego, CA, USA) according to the manufacturer’s instructions. The V3 and V4 hypervariable regions of the bacterial 16S ribosomal RNA gene were amplified by PCR (98°C for 1 min, followed by 30 cycles at 98°C for 10 s, 50°C for 30 s, and 72°C for 30 s and a final extension at 72°C for 5 min) using primers 338F 5′-barcode-ACTCCTACGGGAGGCAGCA-3′ and 806R 5′-barcode- GGACTACHVGGGTWTCTAAT-3′, where barcode is an eight base sequence unique to each sample. All PCR reactions were carried out in 30 μL reactions with 15 μL of Phusion High-Fidelity PCR Master Mix (New England Biolabs); 0.2 μM of forward and reverse primers, and about 10 ng template DNA.

### PCR Products Quantification, Qualification, and Purification

Mix same volume of 1X loading buffer (contained SYB green) with PCR products and operate electrophoresis on 2% agarose gel for detection. Samples with bright main strip between 400 and 450 bp were chosen for further experiments. PCR products were mixed in equidensity ratios. Then, mixture PCR products were purified with GeneJET Gel Extraction Kit (Thermo Scientific).

### Library Preparation and Sequencing

Following manufacturer’s recommendations, sequencing libraries were generated by using NEB Next Ultra DNA Library Prep Kit for Illumina (NEB, USA) and then index codes were added to them. The library quality was assessed on the Qubit 2.0 Fluorometer (Thermo Scientific) and Agilent Bioanalyzer 2100 system. At last, the library was sequenced on an Illumina MiSeq platform and 300 bp paired-end reads were generated. The raw reads were deposited into the NCBI Sequence Read Archive (SRA) database (Accession Number: SRR3186997).

### Processing of Sequencing Data

Paired-end reads from the original DNA fragments are merged by using FLASH software (Magoč and Salzberg, 2011), which is designed to merge paired-end reads when there are overlaps between reads1 and reads2. Paired-end reads was assigned to each sample according to the unique barcodes. Raw sequencing data were quality-filtered using QIIME 1.8.0 software (Caporaso et al., 2010) with the following criteria: (i) The 300 bp reads were truncated at any site receiving an average quality score <20 over a 50-bp sliding window, discarding the truncated reads that were shorter than 50 bp; (ii) exact barcode matching, two nucleotide mismatch in primer matching, reads containing ambiguous characters were removed; (iii) only sequences that overlap longer than 10 bp were assembled according to their overlap sequence. Reads which could not be assembled were discarded. Operational taxonomic units (OTUs) were clustered with 97% similarity cutoff using UPARSE (Edgar, 2013); chimeric sequences were identified and removed using UCHIME (Edgar et al., 2011). Singleton OTUs were removed. The taxonomy of 16S rRNA gene sequence was analyzed by RDP Classifier (Wang et al., 2007) against the Silva rRNA gene database^1^ using confidence threshold of 80%. All samples were normalized at the same sequence depth (12,401 reads) and these OTUs were then used as a foundation for calculating alpha-diversity and beta-diversity metrics using QIIME 1.8.0 software (Caporaso et al., 2010).

### Statistical Analyses

Statistical analyses of the alpha-diversity of each soil sample via Chao1, Good’s coverage estimator, and Shannon’s index (*H′*) were performed using QIIME 1.8.0 software (Caporaso et al., 2010). One-way analysis of variance (ANOVA) followed by Tukey’s HSD (Honest Significant Difference) test was performed for the geochemical properties and the diversity parameters of samples to determine the level of significance using Statistical Package for the Social Sciences software (SPSS) v.17.0. The relationships among the bacterial communities in the 12 samples were analyzed by hierarchical clustering analysis using the R v.3.1.1 statistical software. Analysis of similarities (ANOSIM) test was performed to determine whether the four sampling sites had statistically significantly different bacterial communities by using QIIME v.1.8.0 software (Caporaso et al., 2010). The abundance-based Bray–Curtis similarity coefficient was used to examine the dissimilarity of different samples. The relevance of environmental factors in explaining the distribution patterns of bacterial communities in different samples was analyzed by Bray–Curtis distance-based redundancy analysis (db-RDA) using the R v.3.1.1 statistical software. Monte Carlo permutation test was also performed to examine the relationship between the nine geochemical properties and bacterial community composition in this Arctic lake area. A linear discriminant analysis effect size (LEfSe) method was used to identify the significantly different bacterial groups in different sampling sites (Segata et al., 2011).

## Results

### Geochemical Properties of Soil and Sediment Samples

The highest values of water content, organic C, organic N, ${\mathrm{NH}}_{4}^{+}$-N, ${\mathrm{SiO}}_{4}^{2-}$-Si, and ${\mathrm{PO}}_{4}^{3-}$-P were observed from the sediment samples (site Sedi), whereas the lowest values of pH, ${\mathrm{NO}}_{2}^{-}$-N, and ${\mathrm{NO}}_{3}^{-}$-N were also detected at the site Sedi. Among the nine geochemical properties, six properties (i.e., water content, pH, ${\mathrm{NH}}_{4}^{+}$-N, ${\mathrm{SiO}}_{4}^{2-}$-Si, ${\mathrm{NO}}_{2}^{-}$-N, and ${\mathrm{NO}}_{3}^{-}$-N) were significantly different between sediment samples and soil samples. For examples, the water contents of soils were in the range of 8.68 to 31.22 %, where the water contents of sediments ranged from 48.38 to 56.33%; the ${\mathrm{NH}}_{4}^{+}$-N concentrations in the sediments ranged from 60.00 to 110.22 μg/g, which was much higher than those in the soils (1.48–5.26 μg/g); the concentrations of ${\mathrm{SiO}}_{4}^{2-}$-Si in the sediments were from 32.79 to 68.88 μg/g, which were also much higher than those in soils (3.73–10.12 μg/g). In contrast, the concentrations of ${\mathrm{NO}}_{3}^{-}$-N (0.07–0.19 μg/g) and ${\mathrm{NO}}_{2}^{-}$-N (0.04–0.10 μg/g) in the sediments were lower than those in the soils (${\mathrm{NO}}_{3}^{-}$-N, 0.75–4.20 μg/g; ${\mathrm{NO}}_{2}^{-}$-N, 0.44–1.06 μg/g). Additionally, the other three factors (i.e., organic C, organic N, and ${\mathrm{PO}}_{4}^{3-}$-P) were not significantly different among four samples sites. Specifically, the organic C contents ranged from 0.44 to 11.81%, whereas organic N contents were from 0.06 to 0.94%. The concentrations of ${\mathrm{PO}}_{4}^{3-}$-P were 0–1.71 μg/g. Furthermore, eight geochemical properties (i.e., water content, organic C, organic N, ${\mathrm{NH}}_{4}^{+}$-N, ${\mathrm{SiO}}_{4}^{2-}$-Si, ${\mathrm{NO}}_{2}^{-}$-N, ${\mathrm{PO}}_{4}^{3-}$-P, and ${\mathrm{NO}}_{3}^{-}$-N) were not significantly different among three sampling sites of soils (Hill, Up, and Down).

### Bacterial Diversity and Community Composition

A total of 148,812 bacterial sequences and 2,987 OTUs (at the 3% evolutionary distance) were identified in the present study. The sequence number of each sample was 12,401, from which 1,022 to 1,308 OTUs were recognized. The Good’s coverage estimator of the OTUs in the samples ranged from 95.33 to 97.52% (**Table 2**), indicating that the sequences sufficiently covered the diversity of bacterial populations in all the samples. According to the OTU numbers and Shannon’s indices, no significant differences in bacterial richness and diversity were detected among the four sampling sites (**Table 2**).

**Table 2:** Summary data for Miseq sequencing data from the 12 samples in the present study.

Sample code	Number of sequence	Number of OTUs^#^	Chao 1	Good’s coverage estimator (%)	Shannon’s index
Hill.1	12401	1227	1694	96.38	7.91
Hill.2	12401	1191	1747	96.01	7.04
Hill.3	12401	1046	1363	97.11	7.31
**Average**		**1154**	**1601**	**96.50**	**7.42**
Up.1	12401	1228	1694	96.87	8.17
Up.2	12401	1204	1747	97.28	8.24
Up.3	12401	1308	1361	96.21	8.19
**Average**		**1246**	**1600**	**96.79**	**8.20**
Down.1	12401	1140	1528	96.64	7.36
Down.2	12401	1022	1265	97.52	7.39
Down.3	12401	1301	1695	96.54	8.37
**Average**		**1154**	**1496**	**96.90**	**7.71**
Sedi.1	12401	1100	1361	97.20	7.67
Sedi.2	12401	1211	1600	96.73	8.04
Sedi.3	12401	1245	2552	95.33	7.93
**Average**		**1185**	**1837**	**96.42**	**7.88**

*^#^Defined at the cutoff 3% difference in sequence. Statistical significance was assessed by one-way ANOVA followed by Tukey’s HSD test, and significant differences were accepted when p < 0.05 between the two groups.*

**FIGURE 2:**
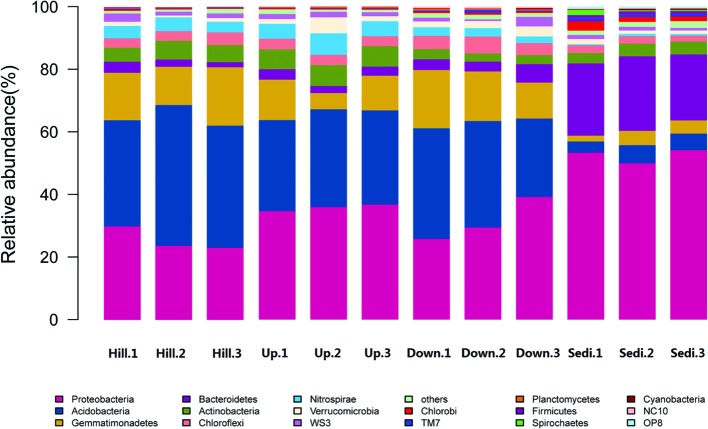
**The relative abundance of different phyla in the 12 soil/sediment samples in the present study**.

Sequences affiliated with Proteobacteria, Acidobacteria, Gemmatimonadetes, and Bacteroidetes were common in all the four sites (**Figure 2**). Proteobacteria and Acidobacteria were abundant phyla in the nine soil samples, whereas Proteobacteria and Bacteroidetes were abundant phyla in the three sediment samples.

The most abundant sequences in Proteobacteria phylum belonged to Betaproteobacteria, followed by Alphaproteobacteria, Deltaproteobacteria, Gammaproteo-bacteria, and Epsilonproteobacteria. Analysis of Proteobacteria revealed dominance of the order Burkholderiales (Class Betaproteobacteria), followed by Sphingomonadales (Class Alphaproteobacteria), Rhizobiales (Class Alphaproteobacteria), Xanthomonadales (Class Gammaproteobacteria), and Syntro-phobacterales (Class Deltaproteobacteria). In Acidobacteria, the most abundant class was Chloracidobacteria. The phylum Bacteroidetes was represented by bacteria belonging to the classes Flavobacteriia, Bacteroidia, and Sphingobacteriia, whereas the abundant order were Bacteroidales (Class Bacteroidia), Sphingobacteriales (Class Sphingobacteriia), and Flavobacteriales (Class Flavobacteriia).

The bacterial genera detected in this study (>1000 reads) included *Zymomonas* (1635 reads, phylum Proteobacteria), *Kaistobacter* (1397 reads, phylum Proteobacteria), *Methylibium* (1340 reads, phylum Proteobacteria), *Sulfuricurvum* (1321 reads, phylum Proteobacteria), *Rhodoplanes* (1278 reads, phylum Proteobacteria), Candidatus *Solibacter* (1141 reads, phylum Acidobacteria), and *Syntrophus* (1098 reads, phylum Proteobacteria).

### The Correlation between Bacterial Communities and Soil/Sediment Geochemical Properties

**FIGURE 3:**
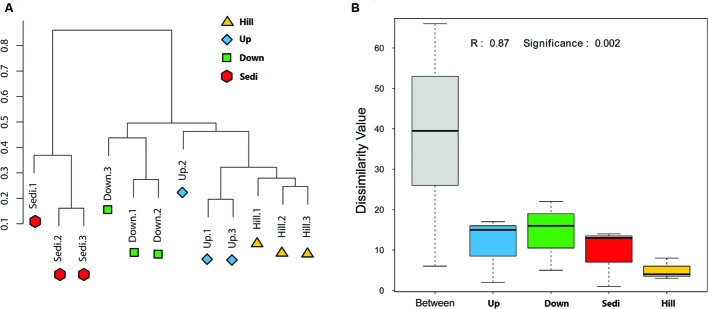
**(A)** Clustering analysis of bacterial communities in the 12 soil/sediment samples based on OTU abundance-based Bray–Curtis similarity coefficients. **(B)** Analysis of similarities (ANOSIM) of the four sampling sites in the present study.

**FIGURE 4:**
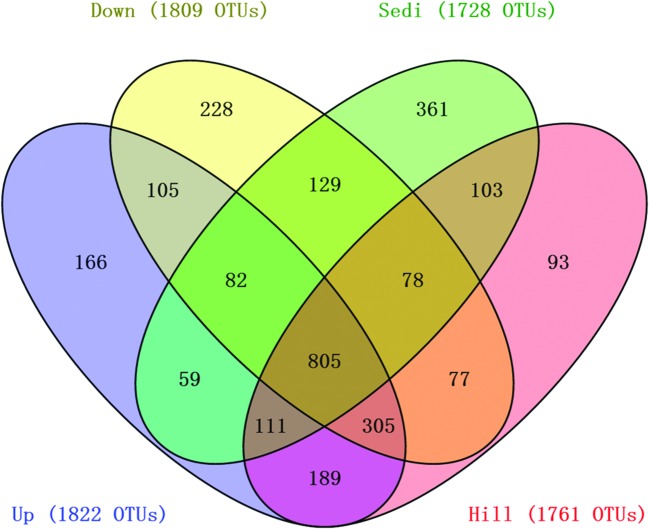
**A Venn diagram displaying the degree of overlap of bacterial OTUs (at the 3% evolutionary distance) among the four sampling sites**.

Operational taxonomic units cluster analysis (**Figure 3A**) revealed that the 12 samples were clustered into four groups which corresponded to four sampling sites very well. It was shown that nine soil samples from sites Hill, Up, and Down were closely related and they were obviously distinguished from three sediment samples from site Sedi. An ANOSIM test (*R* = 0.87, *p* = 0.002) supported that the four sampling sites harbored significantly different bacterial communities (**Figure 3B**). A Venn diagram demonstrated that OTUs differed among the four sites (**Figure 4**). The number of site-specific OTUs ranged from 93 (Site Hill) to 361 (Site Sedi). Only 805 in 2,987 OTUs were shared by all four sampling sites.

At different taxonomic ranks, the four sampling sites can be significantly distinguished from each others, as shown in Supplementary Table S1. For examples, at the phylum rank, members of Chlorobi, Firmicutes, Proteobacteria were significantly abundant in the sediment samples (site Sedi); at the class rank, members of Actinobacteria, Anaerolineae, Clostridia, Ignavibacteria, and Verrucomicrobiae were significantly higher in site Sedi than those in other three sites. Similarly, the bacterial community composition in the four sampling sites can be distinguished at ranks of order, family, and genus. For example, sequences of the genera, such as *Clostridium*, *Luteolibacter*, *Methylibium*, *Rhodococcus*, and *Rhodoplanes*, were significantly different in their abundance among the four sampling sites. Particularly, members of the genera *Clostridium*, *Dechloromonas*, *Desulfomicrobium*, *Haliscomenobacter*, *Hyphomicrobium*, *Luteolibacter*, *Methylibium*, *Mycobacterium*, *Nitrospira*, and *Roseomonas* were significantly abundant in the sediment samples (site Sedi; Supplementary Table S1).

**FIGURE 5:**
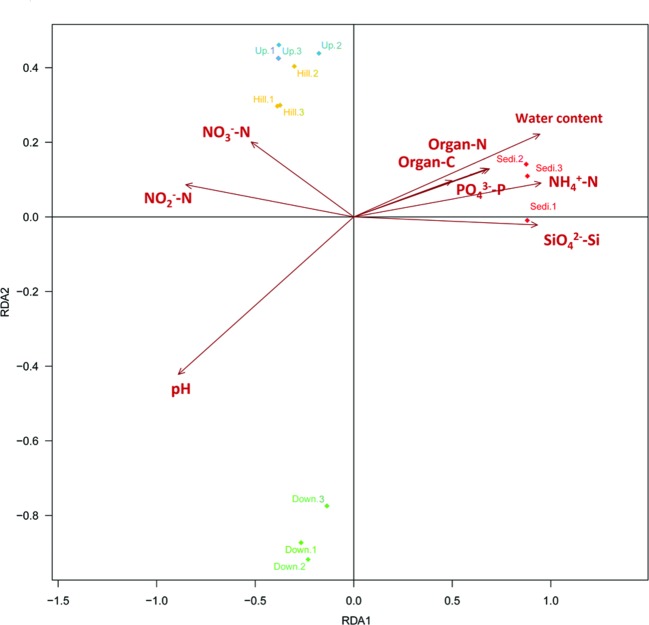
**Distance-based redundancy analysis to show correlations between the bacterial communities and environmental factors of the 12 samples from the four sampling sites.** The arrows represent geochemical factors measured. The 12 samples are labeled with unique sampling code.

Distance-based redundancy analysis (db-RDA; **Figure 5**) and Monte Carlo permutation test (**Table 3**) were performed to examine the relationship between the nine geochemical properties and bacterial community composition in the Arctic lake area. It was also shown that six soil samples from sites Hill and Up were closely related and they were obviously distinguished from soil samples from site Down and three sediment samples from site Sedi. The combination of the nine environmental factors showed a significant correlation with bacterial community composition (*F* = 8.184374, *p* = 0.001). These factors explained 97.36% of the bacterial community variation, while 2.64% of the variation was not explained by any of the selected nine environmental factors. Among the nine geochemical factors, pH (*p* < 0.001), water content (*p* < 0.01), ${\mathrm{NH}}_{4}^{+}$-N (*p* < 0.01), ${\mathrm{SiO}}_{4}^{2-}$-Si (*p* < 0.01), ${\mathrm{NO}}_{2}^{-}$-N (*p* < 0.05), organic C (*p* < 0.05), and organic N (*p* < 0.05) were important factors that correlated with the bacterial community composition in this area. However, the other two environmental factors, including ${\mathrm{PO}}_{4}^{3-}$-P and ${\mathrm{NO}}_{3}^{-}$-N, were not significantly correlated with bacteria community composition (**Table 3**).

**Table 3:** A Monte Carlo permutation test of relationship between environmental factors and bacterial community composition.

	RDA1	RDA2	*r*^2^	*P*-value
Water content	0.973405	0.229093	0.9413	0.003ˆ**
Organic C	0.982851	0.184403	0.4908	0.026ˆ*
Organic N	0.982232	0.187672	0.4737	0.027ˆ*
pH	-0.903472	-0.428648	0.9708	0.001ˆ***
${\mathrm{NH}}_{4}^{+}$-N	0.995426	0.095536	0.9114	0.010ˆ**
${\mathrm{SiO}}_{4}^{2-}$-Si	0.999743	-0.022681	0.8698	0.003ˆ**
${\mathrm{NO}}_{2}^{-}$-N	-0.994949	0.100383	0.7339	0.011ˆ*
${\mathrm{PO}}_{4}^{3-}$-P	0.981501	0.191459	0.2583	0.339
${\mathrm{NO}}_{3}^{-}$-N	-0.932998	0.359882	0.3111	0.147

*^∗^Correlation is significant at the 0.05 level.*

*^∗∗^Correlation is significant at the 0.01 level.*

*^∗∗∗^Correlation is significant at the 0.001 level.*

*P-values based on 999 permutations.*

## Discussion

In the present study, the high level of Shannon diversity indices (*H*′ = 7.04–8.37) and the identification of 1022–1308 OTUs suggest the presence of high richness and diversity of bacterial communities in the soils and lake sediments from Arctic lake area. By comparison, using 454 pyrosequencing, Steven et al. (2013) reported a Shannon diversity of 6.4–7.2 and 1801–2264 OTUs for soil bacterial communities in the catchment area of a lake in the Canadian High Arctic. The observed bacterial richness and diversity indices in this study were somewhat similar to those in the lake area of the Canadian High Arctic.

The relative abundances of the sequences affiliated with Proteobacteria, Acidobacteria, Gemmatimonadetes, and Bacteroidetes in the soils and lake sediments (Svalbard, High Arctic), were somewhat different from those previously described for soils in the Arctic using high-throughput sequencing (Steven et al., 2013). Based on the 16S rDNA pyrosequencing data, Acidobacteria, Cyanobacteria, Proteobacteria, Planctomycetes, and Verrucomicrobia were the dominant soil bacterial phyla in the catchment lake area of the Canadian High Arctic (Steven et al., 2013). Bacterial communities in both organic and mineral soils (at Toolik Lake, Alaska, USA) were predominated by Acidobacteria and Proteobacteria, followed by the Actinobacteria and Bacteroidetes (Campbell et al., 2010). In addition, the dominant sequences in the moist acidic tussock tundra soil in a subarctic region (Northwest Alaska) were Acidobacteria, Proteobacteria, and Actinobacteria (Kim et al., 2014). In the soils collected from Adventdalen (Svalbard), the dominant sequences (>5% of total) were Proteobacteria, Actinobacteria, Verrucomicrobia, Acidobacteria, and Gemmatimonadetes (Schostag et al., 2015). These differences of dominant phyla may be due to the geographical distance and differences in soil properties among the different regions. For example, the carbon contents in this study were in the range of 0.44–11.81%, where the carbon contents in the Canadian High Arctic ranged from 4.25 to 18.97%.

Although bacterial diversity and richness indices did not vary much among the four different sites in this study, some specific bacterial groups varied obviously. For example, members of the genera *Clostridium*, *Dechloromonas*, *Desulfomicrobium*, *Haliscomenobacter*, *Hyphomicrobium*, *Luteolibacter*, *Methylibium*, *Mycobacterium*, *Nitrospira*, and *Roseomonas* were significantly abundant in the lake sediment samples (site Sedi). As significant differences in geochemical properties and bacterial communities were observed between the three soil sites (Hill, Up, Down) and one sediment site (Sedi), the above genera may be specifically associated with the environmental factors in the lake sediments (site Sedi). For example, the carbohydrate-fermenting butyric acid bacteria of the genus *Clostridium* can act as precursors for sulfate reducers and methanogens and thereby perform an important step of anaerobic decomposition of organic matter in aquatic ecosystems (Gorlenko et al., 1977), whereas *Nitrospira* is a chemolithoautotrophic nitrite-oxidizing bacterium that is generally found in freshwater (Hovanec et al., 1998). Therefore, the genera *Clostridium* and *Nitrospira* may be much abundant in the lake sediments as compared with soils near lake.

The combined nine geochemical factors showed a significant correlation with bacterial community composition in this lake area. In this study, pH (*p* < 0.001) was the best predictor of soil bacterial community composition. Furthermore, content of water (*p* < 0.01), ${\mathrm{NH}}_{4}^{+}$-N (*p* < 0.01), ${\mathrm{SiO}}_{4}^{2-}$-Si (*p* < 0.01), ${\mathrm{NO}}_{2}^{-}$-N (*p* < 0.05), organic C (*p* < 0.05), and organic N (*p* < 0.05) showed significant correlation with the bacterial community composition. The above abiotic factors may directly alter bacterial community composition by affecting growth of certain bacteria in soils or lake sediments. In the previous studies, soil pH was found to be the most influential soil properties to determine the bacterial community structure in subarctic tundra soil (Kim et al., 2014; Shi et al., 2015), whereas Steven et al. (2013) reported that soil moisture might influenced the bacterial communities of samples inside and outside of the water tracks in an Arctic lake area. Koyama et al. (2014) found that soil bacterial composition was altered by increased nutrient availability in Arctic tundra soils. Additionally, Shi et al. (2015) found that dissolved organic carbon (DOC), dissolved organic carbon (DON), C/N ratio, ${\mathrm{NH}}_{4}^{+}$ concentration, and N mineralization also contributed to soil bacterial community variability in the subarctic region. In this study, more organic N and ${\mathrm{NH}}_{4}^{+}$-N were found in sediment samples compared to soil samples. Interestingly, the anaerobic bacteria *Clostridium* was also significantly abundant in the lake sediments compared to soil samples, and some *Clostridium* species were capable of fixing free N_2_ into nitrogenous and ammonium compound (Kanamori et al., 1989).

In the future, more and more thaw lakes and ponds would shrink and disappear, as climate warming dries out the freshwater mass in the High Arctic (Vincent et al., 2013). Our results provide the possible consequences of the climate change on the soil geochemical properties and bacterial communities in the High Arctic lake area. If the lake’s water level and size would shrink in the future, the lake sediments would be exposed to air and their geochemical properties would also be altered. As a consequence, the sediment bacterial communities in the lake water (as shown in the Site Sedi) would be changed to those in the soils near lake’s water (as shown in Site Down) and may even to those in the soils far from lake’s water (as shown in Site Up and Hill). Additionally, we will measure methane or CO_2,_ which are accumulated on the soils and sediments and eventually liberated to the atmosphere as a consequence of climate warming. Furthermore, little is known about the presence, diversity, and community composition of Archaea, which may play important roles in biogeochemical cycling in the Arctic soils and sediments. All in all, if we want to determine the relationships between climate change and microbial communities in this lake ecosystem, the long-time survey (10–50 years) on the soil/sediment geochemical properties and microbial communities in this lake area would be necessary in the further study.

## Author Contributions

NW planned the study, collected samples, conducted lab work. TZ wrote the manuscript and conducted parts of lab work. XY, YY, LD, YG, YM, and SW joined in lab work and laboratory analyses. JZ contributed with planning the project and revising the manuscript. All authors reviewed the manuscript.

## Conflict of Interest Statement

The authors declare that the research was conducted in the absence of any commercial or financial relationships that could be construed as a potential conflict of interest.

## Footnotes

1 http://www.arb-silva.de/
