# Supplementary material for: Diversity and Composition of Bacterial Community in Soils and Lake Sediments from an Arctic Lake Area
Source: Front Microbiol. 2016 Jul 28;7:1170. doi: 10.3389/fmicb.2016.01170 (PMC4963411; doi:10.3389/fmicb.2016.01170)
Supplement: Supplementary file 1 [file Table_1.DOCX]

**Table S1 | Lefse analysis showing the taxonomic groups which are significantly different among four sampling sites.**

| Taxonomic rank | Name | Hill | Up | Down | Sedi |
| --- | --- | --- | --- | --- | --- |
| Phylum | Acidobacteria | 5.23 (α=0.038) | - | - | - |
| Phylum | Actinobacteria | - | 4.26(α=0.016) | - | - |
| Phylum | Chlorobi | - | - | - | 3.91(α=0.043) |
| Phylum | Chlorofiexi | - | - | 4.06(α=0.033) | - |
| Phylum | Elusimicrobia | - | - | 3.07(α=0.037) | - |
| Phylum | Firmicutes | - | - | - | 3.63(α=0.029) |
| Phylum | Gemmatimonadetes | 4.76 (α=0.043) | - | - | - |
| Phylum | Nitrospirae | - | 4.39(α=0.016) | - | - |
| Phylum | Planctomycetes | - | - | 3.39(α=0.019) | - |
| Phylum | Proteobacteria | - | - | - | 5.14(α=0.038) |
|  |  |  |  |  |  |
| Class | Acidobacteria |  | 3.08(α=0.041) |  |  |
| Class | Acidimicrobiia |  | 3.71(α=0.023) |  |  |
| Class | Actinobacteria |  |  |  | 3.75(α=0.026) |
| Class | Anaerolineae |  |  |  | 3.58(α=0.034) |
| Class | Chloracidobacteria | 5.11(α=0.024) | - |  |  |
| Class | Clostridia |  |  |  | 3.62(α=0.024) |
| Class | Flavobacteriia |  |  | 3.40(α=0.039) |  |
| Class | Ignavibacteria |  |  |  | 3.76(α=0.032) |
| Class | Nitrospira |  | 4.36(α=0.016) |  |  |
| Class | Phycisphaerae |  |  | 3.35(α=0.019) |  |
| Class | Spartobacteria |  | 3.93(α=0.025) |  |  |
| Class | Termobacula |  |  | 3.03(α=0.024) |  |
| Class | Thermoleophilia |  | 3.94(α=0.025) |  |  |
| Class | Verrucomicrobiae |  |  |  | 3.51(α=0.035) |
|  |  |  |  |  |  |
| Order | Acidimicrobiales |  | 3.64(α=0.022) |  |  |
| Order | Acidobacteriales |  | 3.07(α=0.041) |  |  |
| Order | Actinomycetales |  |  |  | 3.72(α=0.028) |
| Order | Anaerolineales |  |  |  | 3.22(α=0.029) |
| Order | Burkholderiales |  |  |  | 4.62(α=0.032) |
| Order | Caldilineales |  |  |  | 3.23(α=0.029) |
| Order | Chloroflexales |  |  |  | 3.22(α=0.040) |
| Order | Chromatiales |  |  |  | 3.38(α=0.025) |
| Order | Chthoniobacterales |  | 3.96(α=0.025) |  |  |
| Order | Clostridiales |  |  |  | 3.58(α=0.017) |
| Order | Coriobacteriales |  |  |  | 3.23(α=0.038) |
| Order | Desulfovibrionales |  |  |  | 2.98(α=0.043) |
| Order | Desulfuromonadales |  |  | 3.30(α=0.026) |  |
| Order | Entotheonellales |  | 3.29(α=0.028) |  |  |
| Order | Gaiellales | 3.82(α=0.024) |  |  |  |
| Order | Gemmatimonadales |  |  |  | 4.09(α=0.027) |
| Order | Ignavibacteriales |  |  |  | 3.77(α=0.032) |
| Order | Methylomirabiliales |  |  | 3.39(α=0.039) |  |
| Order | Nitrospirales |  | 4.35(α=0.016) |  |  |
| Order | Rhizobiales |  | 4.04(α=0.024) |  |  |
| Order | Rhodocyclales |  |  |  | 4.06(α=0.024) |
| Order | Solirubrobacterales |  | 3.51(α=0.018) |  |  |
| Order | Spirobacillales |  |  |  | 3.36(α=0.042) |
| Order | Syntrophobacterales |  |  |  | 4.38(α=0.025) |
| Order | Termobaculales |  |  | 2.99(α=0.024) |  |
| Order | Verrucomicrobiales |  |  |  | 3.49(α=0.035) |
|  |  |  |  |  |  |
| Family | Acetobacteraceae |  |  |  | 4.41(α=0.049) |
| Family | Alcaligenaceae |  | 3.88(α=0.043) |  |  |
| Family | Anaerolinaceae |  |  |  | 3.18(α=0.029) |
| Family | Brucellaceae |  |  | 3.59(α=0.047) |  |
| Family | Burkholderiaceae |  |  |  | 3.21(α=0.020) |
| Family | Caldilineaceae |  |  |  | 3.22(α=0.029) |
| Family | Chromatiaceae |  |  |  | 3.32(α=0.020) |
| Family | Chthoniobacteraceae |  | 3.93(α=0.025) |  |  |
| Family | Clostridiaceae |  |  |  | 3.49(α=0.028) |
| Family | Comamonadaceae |  |  |  | 4.65(α=0.022) |
| Family | Cyclobacteriaceae |  |  |  | 3.44(α=0.038) |
| Family | Desulfomicrobiaceae |  |  |  | 2.94(α=0.043) |
| Family | Ekhidnaceae |  |  |  | 3.11(α=0.038) |
| Family | Entotheonellaceae |  | 3.25(α=0.028) |  |  |
| Family | Gaiellaceae | 3.85(α=0.023) |  |  |  |
| Family | Geobacteraceae |  |  | 3.30(α=0.026) |  |
| Family | Hyphomicrobiaceae |  | 4.15(α=0.019) |  |  |
| Family | Iamiaceae |  | 2.99(α=0.025) |  |  |
| Family | Ignavibacteriaceae |  |  |  | 4.06(α=0.033) |
| Family | Intrasporangiaceae |  |  |  | 3.09(α=0.038) |
| Family | Koribacteraceae |  | 3.05(α=0.041) |  |  |
| Family | Methylomirobiliaceae |  |  | 3.41(α=0.039) |  |
| Family | Micromonosporaceae |  | 3.22(α=0.041) |  |  |
| Family | Mycobacteriaceae |  |  |  | 3.14(α=0.022) |
| Family | Nigrospiraceae |  | 3.92(α=0.022) |  |  |
| Family | Nocardiaceae |  |  | 3.03(α=0.024) |  |
| Family | Oscillochloridaceae |  |  | 3.53(α=0.033) |  |
| Family | Rhodobiaceae |  | 3.39(α=0.022) |  |  |
| Family | Rhodocyclaceae |  |  |  | 4.05(α=0.024) |
| Family | Saprospiraceae |  |  |  | 3.42(α=0.048) |
| Family | Sinobacteraceae |  |  |  | 4.02(α=0.043) |
| Family | Solirubrobacteraceae |  | 3.04(α=0.031) |  |  |
| Family | Syntrophaceae |  |  |  | 4.29(α=0.044) |
| Family | Syntrophobacteraceae |  | 4.02(α=0.025) |  |  |
| Family | Thermobaculaceae |  |  | 2.99(α=0.024) |  |
| Family | Verrucomicrobiaceae |  |  |  | 3.48(α=0.035) |
|  |  |  |  |  |  |
| Genus | *Afifella* |  | 3.31(α=0.018) |  |  |
| Genus | Candidatus *Methymirabilis* |  |  | 3.44(α=0.039) |  |
| Genus | *Clostridium* |  |  |  | 3.57(α=0.029) |
| Genus | *Dechlorononas* |  |  |  | 3.26(α=0.019) |
| Genus | *Desulfomicrobium* |  |  |  | 2.93(α=0.043) |
| Genus | *Geobacter* |  |  | 3.29(α=0.024) |  |
| Genus | *Haliscomenobacter* |  |  |  | 3.58(α=0.041) |
| Genus | *Hyphomicrobium* |  |  |  | 3.29(α=0.031) |
| Genus | *Iamia* |  | 3.04(α=0.025) |  |  |
| Genus | *Luteolibacter* |  |  |  | 3.48(α=0.037) |
| Genus | *Methylibium* |  |  |  | 3.95(α=0.032) |
| Genus | *Mycobacterium* |  |  |  | 3.10(α=0.022) |
| Genus | *Nitrospira* |  | 3.63(α=0.016) |  |  |
| Genus | *Ochrobactrum* |  |  | 3.65(α=0.046) |  |
| Genus | *Oscillochloris* |  |  | 3.46(α=0.033) |  |
| Genus | *Pedomicrobium* |  | 3.51(α=0.016) |  |  |
| Genus | *Rhodococus* |  |  | 3.05(α=0.024) |  |
| Genus | *Rhodoplanes* |  | 3.89(α=0.019) |  |  |
| Genus | *Roseomonas* |  |  |  | 3.91(α=0.045) |

# LDA score with α value for the factorial Kruskal-Wallis test among sample sites.
